# Blueberry Supplementation Effects on Neuronal and Pathological Biomarkers in Subjects at Risk for Alzheimer’s Disease: A Pilot Study

**DOI:** 10.14283/jarlife.2023.13

**Published:** 2023-08-23

**Authors:** P.M. Doraiswamy, M.G. Miller, C.A. Hellegers, A. Nwosu, J. Choe, D.M. Murdoch

**Affiliations:** 1 Neurocognitive Disorders Program, Department of Psychiatry and Behavioural Sciences, Duke University School of Medicine, Durham, NC, USA; 2 Duke Center for the Study of Aging and Human Development, Duke University Medical Center, Durham, NC, USA; 3 Duke Institute for Brain Sciences, Duke University School of Medicine, Durham, NC, USA; 4 Department of Medicine, Division of Pulmonary, Allergy, and Critical Care Medicine, Duke University School of Medicine, Durham, NC, USA

**Keywords:** Dementia, Alzheimer’s, biomarkers, anthocyanins, longevity, neurons, microglia

## Abstract

**Background:**

There is a need to develop non-invasive practical lifestyle interventions for preventing Alzheimer’s disease (AD) in people at risk, such as those with mild cognitive impairment (MCI). Blueberry consumption has been associated with reduced risk of dementia in some epidemiologic studies and with improvements in cognition in healthy aging adults. Blood-based biomarkers have emerged at the forefront of AD therapeutics research spurred by the development of reliable ultra-sensitive “single-molecule array” assays with 100-1000-fold greater sensitivity over traditional platforms.

**Objective:**

The purpose of this study was to examine the effect of blueberry supplementation in MCI on six blood biomarkers: amyloid-beta 40 (Aβ40), amyloid-beta 42 (Aβ42), phosphorylated Tau181 (ptau181), neurofilament light (NfL), Glial Fibrillary acidic protein (GFAP), and Brain-Derived Neurotrophic Factor (BDNF).

**Methods:**

This was a 12-week, open-label, pilot trial of 10 participants with MCI (mean age 80.2 years + 5.16). Subjects consumed 36 grams per day of lyophilized blueberry powder in a split dose consumed with breakfast and dinner. Baseline and endpoint venous blood was analyzed using an ultrasensitive SIMOA assay. Our aim was to test if blueberry supplementation would particularly impact p-tau181, NfL, and GFAP elevations associated with the neurodegenerative process.

**Results:**

There were no statistically significant (p < 0.05) changes from baseline to endpoint for any of the biomarker values or in the ratios of Aβ42 / Aβ40 and ptau181/ Aβ42. Adverse effects were mild and transient; supplementation was relatively well tolerated with all subjects completing the study.

**Conclusion:**

To our knowledge, this is the first study to prospectively examine the effects of blueberry supplementation on a panel of blood biomarkers reflecting the neurodegenerative process. Our findings raise two possibilities - a potential stabilization of the neurodegenerative process or a lack of a direct and acute effect on beta-amyloid/tau/glial markers. A larger controlled study is warranted.

## Introduction

Worldwide, some 50 million adults have Alzheimer’s disease (AD), and several more million may be at elevated risk for AD by virtue of mild cognitive impairment (MCI) and/or silent accumulation of cortical AD pathology (1-2).

Practical, well-tolerated, and non-invasive lifestyle interventions are highly desirable for AD prevention in at-risk middle-aged and older adults (3-9). Evidence from epidemiological, pre-clinical and pilot clinical studies suggests that regular consumption of blueberries may protect against cognitive decline or dementia (7-17). Blueberries contain high levels of micronutrients and antioxidants (such as anthrocyanins) which in preclinical studies have been found to impact many pathways involved in cognitive function or dementia risk - such as reduced oxidative stress, improved inflammatory response, reversed age-related decrements in cognition, increased cerebral blood flow, enhanced microglial clearance of Aβ, inhibited aggregation of Aβ42, suppressed microglial activation and protection against Aβ-induced neurotoxicity (reviewed in 7, 12-17). Some, but not all, epidemiological studies have found evidence favoring a protective role of blueberry consumption on cognition or AD risk. For example, in the longitudinal Nurses Health Study of 16,010 participants, aged ≥70 years; greater intakes of blueberries was associated with slower rates of cognitive decline – the effect estimates suggested that berry intake delayed cognitive aging by up to 2.5 years (15). Pilot randomized controlled trials have also shown that blueberry consumption improved cognition among older adults with MCI (16, 17).

While the overall body of evidence suggests a beneficial effect of blueberry consumption on AD risk, this has not yet been proven in a large, rigorous, randomized controlled trial. Given the limitations of in-vitro or pre-clinical studies of blueberry mechanisms, there is also a need for additional human biomarker studies to understand whether cognitive benefits arise from antioxidant effects, metabolic effects, anti-inflammatory effects, protection from neurotoxins or through altering other pathways implicated in AD/MCI such as beta-amyloid and tau.

Blood biomarkers have emerged at the forefront of MCI/AD clinical therapeutic development research (1831), spurred by the development of reliable ultra-sensitive “single-molecule array” assays with 100-1000-fold greater sensitivity over traditional platforms. Neurofilament light (NfL) is a marker of neuronal dysfunction whose elevation and rate of change are associated with cognitive decline and hippocampal atrophy (19-23). Studies in some neurodegenerative disorders have also shown that elevated blood NfL levels reduce or stabilize following therapy (22). Likewise, a promising marker of glial activation is GFAP (glial fibrillary acidic protein). There is increased expression of GFAP in reactive glial cells surrounding amyloid plaques and blood GFAP is elevated in AD (24). Other blood markers (Aβ42, Aβ40, t-au, p-tau) reflect neuropathology (18, 25-30). For example, Schindler et al showed that the plasma Aβ42 / Aβ40 ratio predicts current and future brain amyloidosis (18). Park et al found plasma t-tau/ Aβ42 predicted an AD pattern of neurofibrillary tangle deposition (25) as well as longitudinal changes in cerebral amyloid deposition, brain glucose metabolism, and hippocampal atrophy. Overall, the evidence makes a strong case for biomarker-guided pilot trials in AD and MCI (31).

Despite this promising body of evidence, to our knowledge, no clinical study has evaluated the effects of blueberry supplementation on in-vivo pathological and neurodegeneration blood biomarkers in MCI. Such studies would be important to extend findings from in-vitro and pre-clinical studies and rule in or rule out potential mechanisms such as reducing glial activation (GFAP) or reducing axonal degeneration (NfL). Since beta-amyloid and tau are conceptually considered as the hallmarks of MCI due to AD, it would be also be important to know whether blueberry consumption affects these pathways.

This open trial examined the effect of blueberry supplementation on neuronal, glial, and pathology blood biomarkers in 10 subjects with MCI. The blood markers measured were Brain Derived Neurotrophic Factor (BDNF), Neurofilament light (NfL), Glial fibrillary acidic protein (GFAP), Amyloid-beta 42 (Aβ42), Amyloid-beta 40 (Aβ40), and p-tau181. Given the small pilot design of this study, a secondary aim of the study was to examine biomarker performance in a clinical trial setting as to generate better estimates of variance and performance of these assays in a clinical trial representative MCI sample.

## Materials and Methods

### Recruitment, Consent, Inclusion/Exclusion Criteria

The study was approved by the Duke institutional review board, and all participants gave written informed consent prior to participation. The study was registered on clinicaltrials.gov (ID: NCT05172128). Key inclusion criteria were that the subject was between 55-85 years of age, had English-speaking ability, was medically stable, and met criteria for amnestic mild cognitive impairment (impaired delayed verbal recall, normal or near normal overall cognition and function). Early and late MCI diagnosis was based on all available information at intake including history, mental status exam, neurological exams, neuropsychological evaluation (including Wechsler Scale-III Logical Memory and Mini-Mental State Examination), and operationalized using accepted criteria from the national ADNI study. Key exclusion criteria included dementia, significant confounding active neurological/psychiatric disease, unwillingness to restrict consumption of anthocyanin-rich foods, allergy or intolerance to blueberries, significant gastrointestinal disorders or surgery that influences digestion and absorption, history of frequent urinary tract or Clostridium difficile infections, and the presence of unstable, acutely symptomatic, or life-limiting illness.

### Trial Design

Ten participants with MCI were recruited. Participants underwent a two-week “washout” period, where they refrained from consuming anthocyanin-containing foods. Participants were required to complete this two-week washout prior to their baseline blood draw. The following day after the blood draw, the participants began their 12-week blueberry supplementation, maintaining abstention from other anthocyanin-rich foods and drinks. After 12 weeks, participants returned to the clinic for an exit blood draw.

### Blueberry Dosing

Supplementation was in the form of lyophilized blueberry powder packets, mixed with water (18 grams, equivalent to 3/4ths cup of fresh blueberries) with 2 daily meals (36 g/d blueberry powder total; approx. 1.5 servings/d), before breakfast and dinner. The blueberry dose of 36 grams per day in a split dose consumed with meals was based on 1) a 33% increased dose that was previously used in a longer (6-month) trial; 2) a desire to deliver the most effective dose of blueberry bioactives, and 3) a reduced likelihood of any gastrointestinal symptoms. Participants were also instructed to log their times of consumption for compliance monitoring, as well as any inadvertent anthocyanin intake. Blueberry powders were packaged in sealed single-serving packets (18 g/packet) to prevent exposure to light and moisture. Participants were instructed to store packets in home refrigerator to avoid degradation of blueberry bioactives. Nutrient and berry intake function and protocol adherence was assessed by telephone at 1-, 4-, 8-, and 12-week safety calls.

### Quanterix Simoa Ultrasensitive Biomarker Assay

Biomarker assays were done using Quanterix HD-X instrument, a fully automated digital immunoassay platform for biomarker assay testing. The Simoa (Single Molecule Array) technology at the heart of this platform enables the detection and quantification of biomarkers previously difficult or impossible to measure, opening up new applications to address significant unmet needs in life science research. The ultrasensitivity of Simoa assays sets it apart from all other immunoassays available today, offering PCR-like limits of detection with both existing and novel protein biomarkers. The sensitivity of the platform is 1000-fold higher than traditional ELISAs and chemiluminescence and electroluminescence (Luminex, Mesoscale) platforms.

### Plasma Collection

For each participant, peripheral venous blood was drawn in EDTA vacutainers (BD Biosciences, Franklin Lakes, NJ USA). Within 30 minutes after collection, blood was centrifuged at 1800 x g for 10 min to obtain plasma. Plasma was aliquoted in 0.5 mL polypropylene cryotubes and stored at -80°C until the time of assay.

### Plasma Biomarker Assays

Plasma samples were thawed at room temperature and centrifuged at 10,000 x g for 5min prior to assay. Plasma Aβ40, Aβ42, BDNF, GFAP, pTau-181, and NfL concentrations were measured with Simoa (Single Molecule Array) assay kits on an HD-X analyzer (Quanterix, Billerica, MA USA). BDNF (BDNF Discovery Kit, # 102039), pTau-181 (pTau-181 Advantage V2.1 Kit, #104111), and Aβ40, Aβ42, NfL, GFAP (Neurology 4-Plex E Advantage Kit, #103670) kits were assayed according to manufacturer protocol (Quanterix, Billerica, MA USA). All samples were run in duplicate and each assay performed in the same batch. Assays were performed by the same technician blinded to the participant’s state and clinical data. Data were exported from the instrument for biomarker analysis. Subjects were not informed of their biomarker results due to their experimental nature, and this was pre-specified in the consent.

### Severity of MCI

Both early MCI (EMCI) and late MCI (LMCI) were eligible. EMCI was defined by a WMS-III Logical memory delayed recall score of 3-6 with 0-7 years of education, a score of 5-9 with 8-15 years of education, and a score of 9-11 with 16 or more years of education. LMCI was defined by a WMS-III Logical memory delayed recall score ≤ 2 with 0-7 years of education, a score ≤ 4 with 8-15 years of education, and a score ≤ 8 with ≥ 16 years of education. Other inclusion criterion was a Folstein Mini-Mental State Examination score of ≥ 23/30.

### Dietary Assessment

At the initial screening visit, subjects completed a dietary questionnaire, as well as a diet history questionnaire, for berry consumption during the past year. Subject diaries were used to check for compliance during the study.

### Safety

Adverse events (AEs) were monitored and serious AEs were reported to the IRB as appropriate.

### Statistical Analysis

Statistical analysis was done using Stata/MP 13.0 and RStudio Version 2022.12.0. The effect of blueberry supplementation on change from baseline in biomarker levels was assessed via paired t-tests. Posthoc responder analyses were performed to compare participants who experienced an increase or decrease in specific biomarkers. Pearson’s correlation coefficients were calculated to test for associations among baseline biomarker measurements. A correlation network was created amongst key baseline and endpoint variables.

## Results

**Figure 1.**
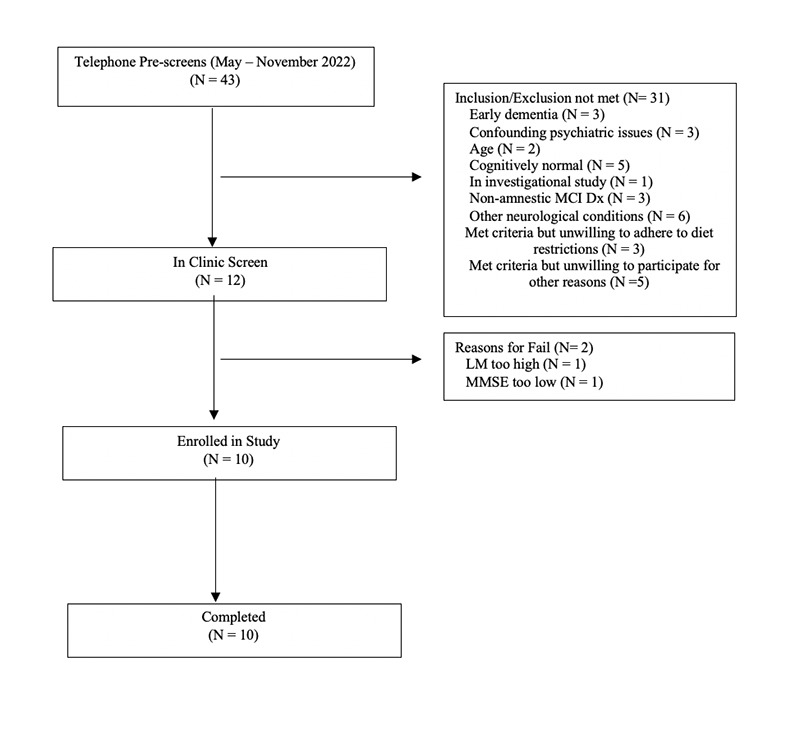
Consort Diagram

Forty-three individuals were prescreened for the study. Of these, twelve participated in in-clinic screening, with ten being eligible for enrollment (Figure 1). Table 1 shows the baseline characteristics. The mean age was 80.2 years (SD 5.16). While the study was open to all genders, the first ten participants who met enrollment criteria were male. No outcome data were excluded. Following initial neuropsychological testing administered by the study site coordinator, nine subjects were classified as having LMCI and one with EMCI.

**Table 1.** Baseline characteristics of the study subjects

Variable	Total
Sample size (N)	10
Age (years) (mean ± sd)	80.20 ± 5.16
Education (years) (mean ± sd)	18.10 ± 2.15
LMCI / EMCI	9/1
MMSE Total (mean ± sd)	27.20 ± 2.15
Wechsler Logical Memory Delay (mean ± sd)	4.80 ± 3.43
Stable Donepezil therapy	3
Stable memantine therapy	2
MIND Diet score at screening (mean ± sd)	8.9 ± 2.73
BMI (mean ± sd)	25.7 ± 2.31
% with cardiovascular disease (e.g. high cholesterol, HTN, A-fib, Hx of MI, CAD)	90%
% with metabolic disease (e.g. hypothyroidism, Type 2 DM)	50%
Blueberry consumption of less than ¼ cup per serving anytime in the past 12 months	80%
Blueberry consumption of more than ¾ cup per serving anytime in the last 12 months	0%
Other Berry Fruit consumption of any amount in the last 12 months	100%

### Effect of Blueberry Supplementation on Biomarker Outcomes

**Figure 2.**
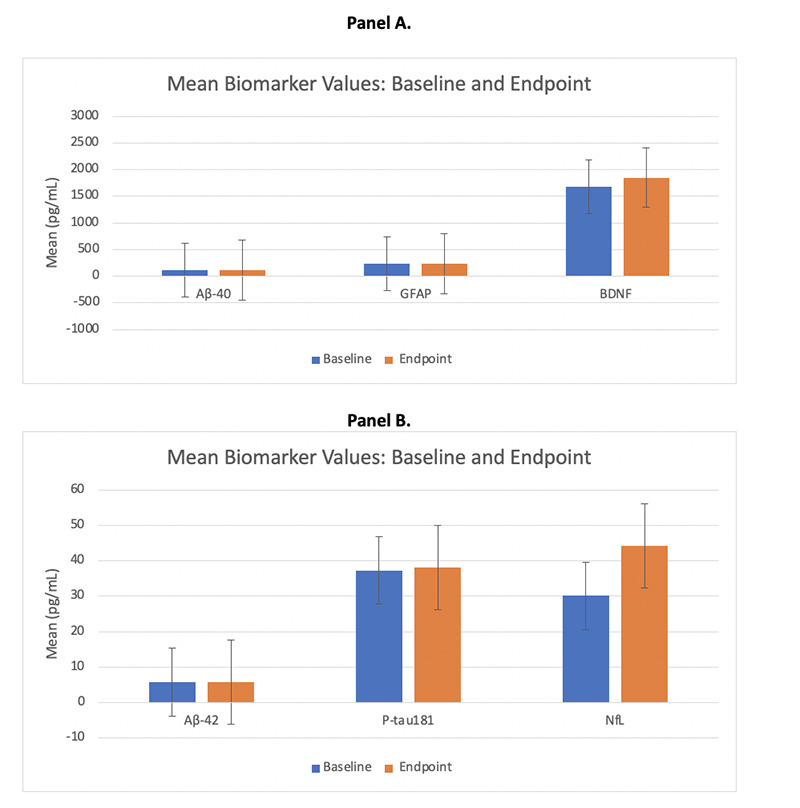
Effect of Blueberry Supplementation on Blood Biomarkers

There were no statistically significant (p < 0.05) changes from baseline at 3 months in any of the biomarker values (Table 2, Figure 2). Ratios of biomarkers also did not significantly change from baseline to 3 months following supplementation.

Figure 2A and Figure 2B depict mean (SD) plasma biomarker values at baseline (depicted in blue) and week 12/endpoint (depicted in orange). There were no statistically significant differences for any of the biomarkers measured. Abbreviations: Aβ40 = amyloid-beta 40; Aβ42 = amyloid-beta 42; ptau181 = phosphorylated Tau181; NfL = neurofilament light; GFAP = Glial Fibrillary acidic protein; BDNF = Brain Derived Neurotrophic Factor

**Table 2.** Effect of Blueberry Supplementation on Neuronal and Pathological Biomarkers (mean ± SE)

Biomarker	Baseline	Post-Treatment	p-value
Aβ40 (pg/mL)	114.45 ± 4.67	114.73 ± 4.27	0.55
Aβ42 (pg/mL)	5.75 ± 0.42	5.75 ± 0.36	0.50
Aβ42 / Aβ40 ratio	0.050 ± 0.002	0.050 ± 0.002	0.51
ptau181 (pg/mL)	37.24 ± 3.31	38.04 ± 2.58	0.64
ptau181/ Aβ42 ratio	6.72 ± 0.65	6.79 ± 0.56	0.56
NfL (pg/mL)	30.09 ± 4.64	44.25 ± 16.85	0.80
GFAP (pg/mL)	239.86 ± 31.15	234.27 ± 26.98	0.34
BDNF (pg/mL)	1685.48 ± 480.43	1850.15 ± 436.63	0.61

1SE, standard error; Aβ40, amyloid-beta 40; Aβ42, amyloid-beta 42; ptau181, phosphorylated Tau181; NfL, neurofilament light; GFAP, Glial Fibrillary acidic protein; BDNF, Brain Derived Neurotrophic Factor

### Biomarker Responder Analyses

Post-hoc responder analyses showed that those who experienced an improvement in NfL values following supplementation tended to be older. Five subjects each showed numeric improvements in NfL or GFAP or pTau181. Two subjects showed numeric improvements in both NfL and GFAP and in both NfL and pTau181.

### Correlations amongst Biomarker and Cognitive Variables

**Figure 3.**
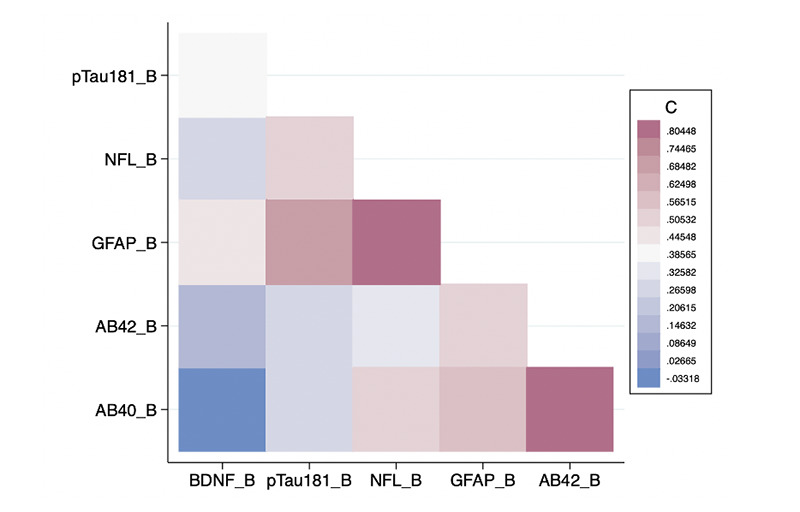
Heat Map of Baseline Biomarker Correlations

**Figure 4.**
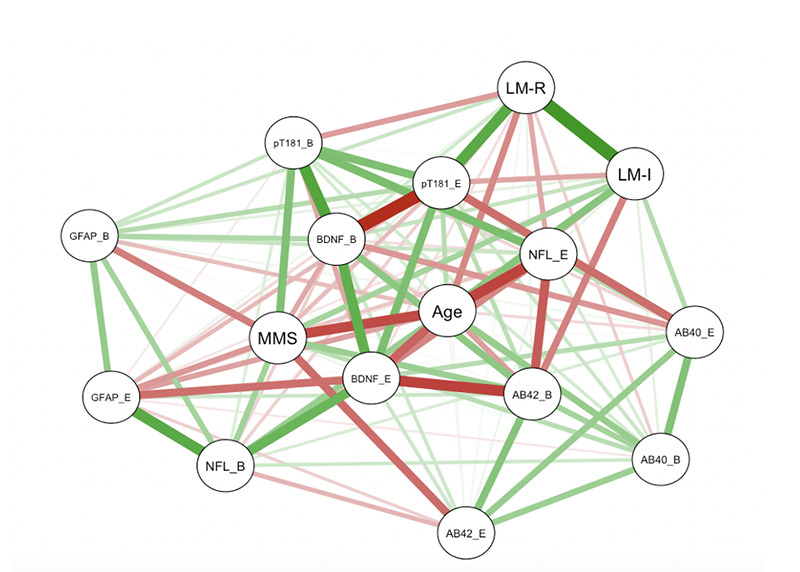
Correlation Network of Cognitive and Biomarker Variables in MCI

As shown in Figure 3, there was a significant positive correlation between baseline GFAP and NfL levels (r = 0.8026; p = 0.0052) as well as between baseline Aβ42 and Aβ40 levels (r = 0.8344; p = 0.0027) (Figure 3). There was a significant negative correlation between MMSE and baseline GFAP (r = -0.6417; p = 0.0455), as well as endpoint GFAP (r = -0.7103; p = 0.0213) (Figure 4). These correlations were expected, and support the validity of the assays.

Figure 3 shows a correlation heat map among the baseline biomarker values for MCI subjects. The index on the right shows the strength of the correlations. The ones in darker colors depict stronger correlations. Abbreviations: Aβ40_B = amyloid-beta 40 at baseline; Aβ42_B = amyloid-beta 42 at baseline; pTau181_B = phosphorylated Tau181 at baseline; NfL_B = neurofilament light at baseline; GFAP_B = Glial Fibrillary acidic protein at baseline; BDNF_B = Brain Derived Neurotrophic Factor at baseline

Figure 4 depicts a correlation network amongst age, baseline cognition (MMSE and Logical Memory Immediate and Delayed), baseline plasma biomarker variables, and endpoint plasma biomarker variables. The green lines depict positive correlations, and the red lines depict negative correlations. The thickness of the lines connecting the nodes depicts the strength of the correlations. Please see the text for details. Abbreviations: Aβ40_B = amyloid-beta 40 at baseline; Aβ40_E = amyloid-beta 40 at endpoint; Aβ42_B= amyloid-beta 42 at baseline; Aβ42_E = amyloid-beta 42 at endpoint; pTau181_B = phosphorylated Tau181 at baseline; pTau181_E = phosphorylated Tau181 at endpoint; NfL_B = neurofilament light at baseline; NfL_E = neurofilament light at endpoint; GFAP_B = Glial Fibrillary acidic protein at baseline; GFAP_E = Glial Fibrillary acidic protein at endpoint; BDNF_B = Brain Derived Neurotrophic Factor at baseline; BDNF_E = Brain Derived Neurotrophic Factor at endpoint; LM-I = Logical Memory-Immediate; LM-R = Logical Memory-Recall; MMS = Mini Mental Status Examination

### Variance Estimates for the Biomarkers

Table 2 presents the variance (SD) estimates for the biomarkers before and after treatment.

### Adverse Events

Supplementation was relatively well tolerated, and all subjects completed the study. Observed adverse events were largely mild and transient. One subject had constipation and stool changes at week 3 which resolved after a week of withholding. One subject had mild constipation which resolved on its own. One subject had transient diarrhea at week 12 which resolved on its own. One subject reported a single episode of abdominal pain at week 10 which resolved on its own. One subject had a scalp abrasion and ankle sprain from a fall but recovered fully. One person had hand tremors and lightheadedness at week 2 which lasted just a day and resolved on its own. One subject was hospitalized overnight for altered mental status (on Day 6) but was ruled out for stroke with brain imaging. Following discharge, this subject had full resolution of symptoms, and this event was not considered to be study related. A few months later, this subject also experienced possible hematemesis (weeks 8 -9) attributed to his pre-existing GERD and Barrett’s esophagus, and it resolved on its own. At Week 12, this subject also developed a COVID-19 infection, and their exit visit was delayed until the infection was resolved. The infection was not considered to be supplement related.

### Compliance

Compliance (% doses) for blueberry powder consumption and adherence to the requirement to avoid other sources of dietary berries was high (>90%). Two subjects withheld dosing transiently for adverse effects as noted above.

### Concomitant cognitive enhancer medications / vaccines

One subject started donepezil at week 1 and also received a COVID-19 vaccine at week 10 both at his doctor’s recommendation.

## Discussion

To our knowledge, this is the first pilot study to examine the effects of blueberry supplementation on neuronal, glial, and pathology blood biomarkers in subjects with MCI. The blood markers measured were neurofilament light (NfL), glial fibrillary acidic protein (GFAP), Aβ42, and p-tau181. The former are markers of neuronal and glial function whereas the latter are markers of pathological changes. As stated previously, biomarkers have come to the forefront of neurodegenerative disorders therapeutic research (18-31) with the US FDA having given accelerated approval to two immunotherapies for Alzheimer’s and one drug for ALS based on reduction in pathological or neurodegenerative biomarkers, respectively.

Blueberry supplementation has been shown in some prior studies to benefit cognitive aging and preclinical studies have reported that blueberry bioactives may provide anti-oxidant benefits, enhance microglial clearance of Aβ, inhibit aggregation of Aβ42, or suppress microglial activation and provide protection against Aβ-induced neurotoxicity (reviewed in 7-17). Blueberry supplementation has also been reported to improve cognition in pilot studies of MCI (16, 17). However, no prior study had examined blueberry supplementation effects on in-vivo biomarkers of neurodegeneration, glial activation as well as amyloid or tau pathways in MCI patients at risk for Alzheimer’s.

In our study, over the 3 months of blueberry supplementation, the MCI subjects did not experience any significant change (decline or improvement) in neuronal, glial, and pathological blood biomarkers. There are two possible interpretations of this finding. The natural course of MCI, especially late MCI, is usually that of progressive neurodegeneration accompanied by elevations of NfL, GFAP, and pTau181 (25-27); hence, there is a possibility that blueberry supplementation stabilized this process. The alternate interpretation is that blueberry supplementation does not acutely (over 3 months) impact beta-amyloid or tau pathways and/ or that it’s potential neuroprotective effects may occur through other pathways such as through reduced oxidative stress, improved inflammatory response, and/ or increased cerebral blood flow.

The strengths of our study are the careful selection of MCI subjects, the relatively high compliance with diet and the use of sensitive state-of-the-art biomarker assays to measure neuronal, glial, and pathological changes.

Limitations of the study include a small sample size, relatively short duration of dosing, and lack of a control group. While direct examination of brain changes using PET scans or obtaining cerebrospinal fluid biomarkers may be more optimal than blood biomarkers, such studies are more invasive and very expensive. In addition, future studies should also examine the long-term performance effects of dosing on blood biomarkers and how measures will vary. This work should be conducted in different geographical areas among diverse populations. It should also be noted that all subjects had consumed some form of berry fruit within in the last 12 months prior to their enrollment, although 80% of subjects consumed less than ¼ cup per time of consumption. It is difficult to get blueberry naïve subjects for such a trial. Last but not least, two biomarkers in our panel (Aβ42 and pTau) were selected on the assumption of cortical beta-amyloid and tau as a causal driver for cognitive impairment in MCI/AD; future research should also examine the conceptual basis for cognitive impairment in MCI driven by other factors, such as calcium regulation, metabolism, vascular risks, or inflammatory pathways independent of and/or upstream of beta-amyloid and tau’s effect on cognitive impairment. Such research should also examine other markers of neuronal dysfunction as well as the antioxidant role of blueberry supplementation.

Despite these limitations, the observations and biomarker variance estimates from this study may help guide the design of future randomized trials. Given the possible need for longer follow-up time, as well as the need for a larger sample size in prevention trials, future clinical research should explore the role of pragmatic trial designs such as “randomized group design” where the randomization unit is no longer an individual, but a cluster or group that could increase trial conduct efficiency.

In summary, to our knowledge, this is the first study to prospectively examine the effects of blueberry supplementation on a panel of blood biomarkers reflecting the MCI/AD neurodegenerative process. Our findings warrant replication in a larger controlled study over a longer period.
